# A Center of a Different Stripe

**DOI:** 10.1289/ehp.113-a160

**Published:** 2005-03

**Authors:** Julia R. Barrett

What do tiny black-and-white-striped zebrafish have in common with human beings? As it turns out, quite a lot. The zebrafish and other aquatic model organisms are helping researchers to better understand complex biomedical questions including how humans develop and are affected by toxic exposures. This work is the focus of the and Freshwater Biomedical Sciences Center (MFBSC) at the University of Wisconsin (UW) –Milwaukee, an NIEHS environmental health sciences center.

“Most investigators who work with living systems use mammalian species as model systems,” says director David Petering, a professor of chemistry at UW–Milwaukee. “So, up until recently, general toxicologists wondered what in the world aquatic organisms could contribute to the study of environmental health and toxicology. It turns out that, historically, aquatic organisms have been enormously important in the development of biomedical research.” Petering notes, for example, that most of the basic understanding of how nerve impulses are conducted arose from studies of the large nerve axon of the squid.

The MFBSC is one of four aquatic research centers funded by the NIEHS. It is located in the Wisconsin Aquatic Technology and Environmental Research (WATER) Institute at UW–Milwaukee. (In addition to UW–Milwaukee faculty, the center draws researchers from several other Wisconsin institutions, including Marquette University, the Medical College of Wisconsin, and UW–Madison.) The center’s genesis dates to the late 1970s, when former NIEHS director David Rall promoted the use of aquatic systems to study human and environmental health issues. The Milwaukee center was the first MFBSC to form. It has since evolved into an advanced aquatic animal facility with laboratories supporting four core research areas: signal transduction and endocrine disruption, metals toxicology and neurotoxicology, the use of zebrafish as a model organism for developmental toxicology, and behavioral toxicology. Additionally, the MFSBC supports a pilot project program, which offers researchers an opportunity to explore the utility of aquatic models in environmental health research and to further develop methods that are already in use.

## The Zebrafish Initiative

Petering says center staff decided within the past decade to focus heavily on developing the zebrafish as a model for developmental toxicology. The zebrafish, a small tropical fish found in many home aquaria, is an attractive developmen-toxicology model for several reasons. To start, development occurs externally, and the embryo is clear. “You can literally watch the entire process of development at a microscopic level,” says Petering. Another appealing feature is that basic development occurs within days, a very rapid time frame compared with mammalian systems, and one that allows researchers to conduct multiple experiments within a short period of time. Also, the rapid development—paired with being able to have a large number of fertilized eggs at once—permits mutagenesis studies (because mutagenesis is a relatively rare event, it’s necessary to have many individual test organisms to detect mutations and determine whether or not they are random events). Finally, zebrafish are very small and easy to maintain.

Richard Peterson, a center investigator and UW–Madison professor of pharmacy, vouches for the usefulness of zebrafish. According to Peterson, who has done extensive pioneering work with lake trout and other Great Lakes and local fish species, zebrafish are ideal for modeling development. “There’s a lot known about the genetics of zebrafish, and there’s a lot known about the developmental biology of the zebrafish,” he says. “In the zebrafish we know what normal is—we know what normal development looks like, which genes are involved, and we know a lot about various genes in terms of their function.”

Peterson’s colleague, Warren Heideman, also a UW–Madison professor of pharmacy, further notes that the zebrafish being a vertebrate bolsters its value as a model species. “A lot of genetic systems that people use, such as *Drosophila* [the fruit fly], nematodes, or yeast, are very powerful for identifying important genes and tracing signaling mechanisms, but they’re not vertebrates, so one wonders how much one can extrapolate the results from, say, a worm to those of a human,” he says. “Zebrafish have taken us a step farther because they are vertebrate organisms, and we expect to find things more similar in zebrafish to humans.”

One of the ways in which Peterson and Heideman are currently using the zebrafish model is to investigate the effects of dioxin exposure on cardiac development. “Not only are fish extremely sensitive to dioxin during early life stage development, we have also determined that the heart is an important target organ for dioxin developmental toxicity,” says Peterson.

Heideman and Peterson recently embarked on a collaborative project to investigate in greater detail the underlying mechanisms of this toxicity in zebrafish. The researchers hope to identify the genes involved as well as the various adverse morphological, functional, biochemical, and cellular effects of dioxin. Peterson says the knowledge gained through these studies should be applicable to other species where the cardiovascular system has been shown to be adversely affected by dioxin—eventually including humans.

## Uncovering Gene–Environment Links

The center’s focus on zebrafish fosters other research methods, notes Petering. “Another strength of the center that has come along with the zebrafish initiative is a strong emphasis on genetics,” he says. This emphasis is apparent in the center’s involvement with the NIEHS-initiated Environmental Genome Project, a multi-component study of more than 500 genes suspected to be environmentally responsive and likely to have a role in regulating environmental diseases.

Michael Carvan, an assistant scientist at the WATER Institute, is part of a group working to identify environmentally relevant genes in the zebrafish. “We’re looking at whether these genes are involved in different environmentally induced disease processes in zebrafish, primarily developmental effects and developmental toxicity,” he says. Another goal is to compare the structures of the genes in the zebrafish with those in humans. “Hopefully, we’ll be able to find certain parts of the gene that are highly conserved between zebrafish and humans—that will give us more evidence that the zebrafish is a good model for humans,” Carvan says.

Another area of research focuses on environmental chemicals and the mechanisms by which they produce developmental abnormalities if significant exposure occurs. Carvan and his colleagues have used genetically distinct strains of zebrafish to identify candidate genes that may influence the response to toxic exposures. Utilizing a cDNA microarray developed at the center, researchers can test numerous end points. “We’re correlating the changes in gene expression in the embryos and larvae to changes in behavior, changes in learning and memory, changes in mortality, and changes related to the development of [effects such as] craniofacial abnormalities,” says Carvan.

For example, human and animal data suggest that a strong genetic component determines sensitivity to ethanol and development of fetal alcohol syndrome. “We’ve shown that early exposure to ethanol causes learning disabilities,” says Carvan. When zebrafish larvae were exposed for the first 24 hours after fertilization to alcohol concentrations comparable to those below the legal driving limit, significant learning disabilities resulted in adult zebrafish. This work is described in the November–December 2004 issue of *Neurotoxicology and Teratology*.

When studying subtle craniofacial malformations, the researchers again found evidence for low-level effects of alcohol exposure. “At quite low levels of alcohol we found significant changes in the structure of the face and the head of the larval zebrafish, showing that they are very sensitive to alcohol-induced developmental abnormalities much like human children as well as mice, rats, and other experimental animals are,” says Carvan. Work is continuing on uncovering the mechanism by which this damage occurs.

## A Behavioral View

Petering notes that for all the emphasis on cellular and molecular effects of environmental toxicants, the center also strongly supports looking at the overall picture of the whole organism. He explains: “A lot of the work that you immediately think of in terms of mechanisms of toxic chemicals in zebrafish and other organisms focuses down on the cellular and molecular effects. But if you don’t know that you actually have an organismic effect, then the rest of it may or may not be significant.” Defining this organismic effect is the mission of the center’s recently created behavioral toxicology core. Currently eight center-related researchers are either actively involved in integrating behavioral tests into their own research or are planning to do so.

Daniel Weber, an assistant scientist at the MFBSC, says the foundation for the behavioral toxicology core was laid about 15 years ago. At that time, Weber was completing doctoral work in behavioral toxicology at UW–Milwaukee by looking at the effect of lead exposures on juvenile and adult fathead minnows. Behavioral outcomes included reproductive and feeding behaviors, simple learning, and other behaviors such as swimming capacity.

Weber and colleagues found they could use smaller concentrations for shorter periods (3–7 days) to observe effects similar to those seen in humans, such as hyperactivity, decreased muscle coordination, learning deficits, changes in energy use, and lower reproductive activity. Use of behavioral toxicology methods led to a collaboration with the Wisconsin Department of Natural Resources and the U.S. Geological Survey to evaluate the effect of urban stormwater runoff on water quality. The team found reproductive behavior and success to be a more sensitive end point than fish mortality for measuring chemical contamination in urban streams.

After completing the program, Weber and his colleagues continued doing behavioral experiments, focusing in on how lead exposure can alter circadian rhythms and reproductive behaviors. The next step was developing a model to investigate the underlying physiologic mechanisms, and the researchers began looking at reflex reactions in fish, such as swimming away when someone taps on the side of the tank. Such reactions involve a simple nerve pathway controlling a specific behavior. Should there be any change in the behavior, it’s a relatively easy task to examine the nerve pathway to identify where the damage has occurred.

Weber and his group have focused for a number of years on different kinds of stimuli to create reflex reactions. “For example,” he says, “we have looked at sound, vibration response, and visual response, and looked at ways in which to measure the kinds of changes that might occur.”

For all the simplicity of the model, though, data collection requires highly sensitive equipment including the capacity for high-speed photography. This allows the researchers to analyze how the animal is moving, its response time, and many other reaction variables. “The reflex reaction is over in about ten milliseconds,” says Weber. “If you use regular photography, it’s just not going to [capture the reaction]. The digital cameras that we use are able to record the equivalent of five hundred frames per second. This information is downloaded into the computer and with the software we have, we can analyze the movements frame by frame.”

This capacity for investigating behavioral toxicology is currently being applied to the environmental health problem of methylmercury-contaminated fish in the Great Lakes. For the past 12 years, center researcher John Dellinger has been working with Native American tribes in northern Wisconsin, trying to analyze the effect of dietary methylmercury exposure among these populations. As part of this work, Weber is helping develop a model in which fish, primarily females, receive dietary methylmercury exposures comparable to those of the tribe members. Subsequent offspring of the fish will be examined throughout their life with regard to learning, reflex response, and other behaviors.

As an additional component to this research, investigators will be looking at whether certain dietary factors can ameliorate the toxicity of methylmercury. This project is being done in conjunction with the Great Lakes Native American Research Centers for Health, a collaborative effort involving the Great Lakes Intertribal Council, several UW campuses, the Mayo Clinic, and the Wisconsin Indian Education Association. It will focus on the role selenium may play in preventing some methylmercury-related neurotoxicity.

“What we’ve seen so far is that adding a little bit of selenium does reduce the embryotoxic effects [of methylmercury],” says Carvan, the principal investigator on the project. “So we’re using microarrays to figure out exactly what’s going on, how the selenium is ameliorating the toxic effects.”

Carvan notes that Native Americans in the Great Lakes region eat a lot of wild rice, which happens to be high in selenium. “If they eat the mercury-contaminated fish, they’re getting all the known benefits of eating fish, but they’re also getting the bad stuff that goes along with mercury,” he says. “If they supplement their diet with wild rice, so that they’re getting more selenium, perhaps that’s going to reduce the toxic effects of the mercury on their offspring.” The methods created for this research will also be expanded to include other fish species as well as other types of contaminants such as ethanol and organophosphate pesticides.

“In terms of where we’re going in the future, you’re going to see more use of models like the zebrafish than you have ever seen in the past,” Peterson says. He envisions a future where very simple models such as zebrafish and *Caenorhabditis elegans* are used instead of mice and rats in low-cost chemical screening. “There’s going to be an important position for the zebrafish in toxicity testing in the future,” he predicts. “We’re only now beginning to become aware of the various ways that this model can be used.”

## Figures and Tables

**Figure:**
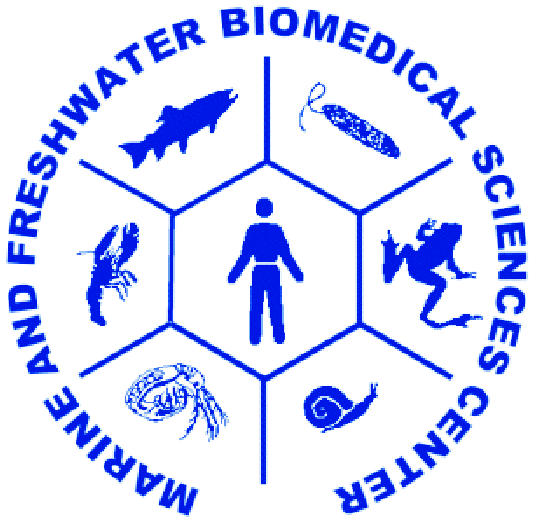


**Figure:**
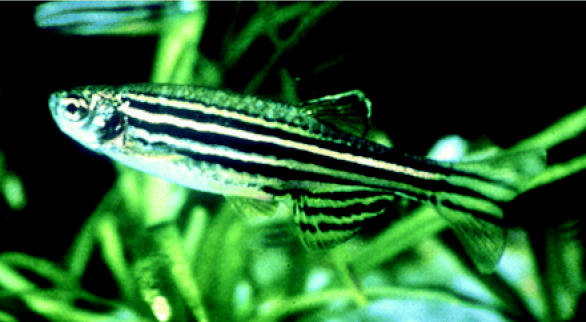
**Small wonder.** The tiny zebrafish is proving to be a giant advantage to researchers studying neurotoxicity and development in humans.

**Figure:**
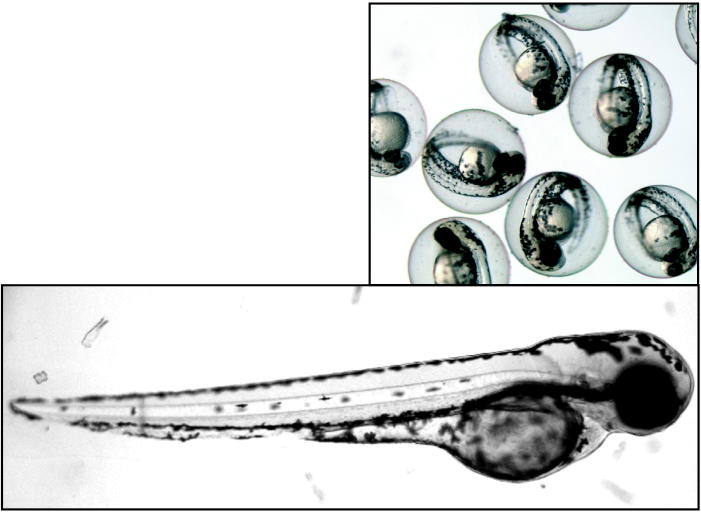
**The inside scoop.** Because zebrafish development occurs externally and the embryo is clear, scientists can literally watch development as it happens. Zebrafish develop from egg (top) to embryo (above) to adult in about three days, allowing fast results from experiments.

**Figure:**
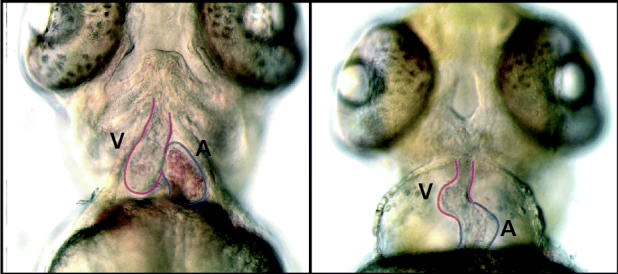
**Dioxin and development.** (left) A ventral view of a control zebrafish embryo 72 hours postfertilization shows the atrium (A) and ventricle (V) of the heart. (right) A 1-hour exposure to 1 part per billion TCDD in the water just after fertilization—yielding tissue concentrations in the parts-per-trillion range—results in malformations of the heart and head. This kind of work takes three days to complete in zebrafish and up to six months to complete in some of the salmonids affected by dioxins and PCBs in the United States.

